# Benign Metastasizing Leiomyoma of the Lung: Diagnostic Process and Treatment Based on Three Case Reports and a Review of the Literature

**DOI:** 10.3390/biomedicines10102465

**Published:** 2022-10-02

**Authors:** Małgorzata Edyta Wojtyś, Olga Kacalska-Janssen, Konrad Ptaszyński, Piotr Lisowski, Michał Kunc, Janusz Wójcik, Tomasz Grodzki

**Affiliations:** 1 Department of Thoracic Surgery and Transplantation, Pomeranian Medical University in Szczecin, 70-891 Szczecin, Poland; 2 Department of Gynecological Endocrinology, Jagiellonian University Medical College, 31-501 Krakow, Poland; 3 Department of Pathology, Faculty of Medicine, Collegium Medicum, University of Warmia and Mazury in Olsztyn, 10-561 Olsztyn, Poland; 4 Students’ Scientific Circle of the Department of Thoracic Surgery and Transplantation, Pomeranian Medical University in Szczecin, 70-891 Szczecin, Poland; 5 Department of Pathomorphology, Medical University of Gdansk, 80-214 Gdańsk, Poland

**Keywords:** benign metastasizing leiomyoma, lung, surgery, pulmonary nodule, case report

## Abstract

Uterine leiomyomas may occasionally spread to the lungs forming nodular lesions detectable on chest X-ray. This condition known as benign metastasizing leiomyoma (BML) usually occurs in females with a history of hysterectomy or myomectomy. We present three cases of BML demonstrating the diagnostic process and treatment approaches. Two patients presented with the more common multiple-nodule variant while the other had a single mass, but all were symptom-free. The age of presented patients at diagnosis of BML ranged from 46–53. The first patient was diagnosed with BML at the age of 50, and 12 years prior to the diagnosis, underwent a supracervical hysterectomy. The second patient had a myomectomy at 36, and BML was diagnosed 17 years later at the age of 53. The third patient had a hysterectomy with bilateral salpingo-oophorectomy at the age of 46, with lung lesions present before the hysterectomy. Immunohistochemical studies of postoperative materials showed positive staining of spindle cells with antibodies against desmin and smooth muscle actin, as well as estrogen and progesterone receptors. The final histopathological diagnoses were pulmonary BML. All patients are stable and symptom-free: two at two years follow-up and one at six months follow-up

## 1. Introduction

Uterine leiomyomas (also referred to as fibroids or myomas) are the most common pelvic tumors in females [1]. They are benign neoplasms arising from the smooth muscle cells of the myometrium. They occur in reproductive-age women and are often asymptomatic [2]. However, when symptomatic, the primary symptoms include menorrhagia with subsequent anemia, abdominal pressure, abdominal pain, urinary frequency, and infertility related to the volume and location of the tumor. Treatment strategies are typically individualized based on the severity of the symptoms, the size and location of the fibroids, the patient’s age, and the patient’s desire for future fertility. The usual goal of therapy is to relieve symptoms. The gold standard of leiomyoma treatment is surgical intervention. Hysterectomy is the definitive surgical procedure, but myomectomy is commonly performed, especially in women who desire to remain fertile. More recently developed techniques, such as uterine artery embolization, are sometimes recommended as minimally invasive procedures. Systemic therapy such as administering of gonadotropin-releasing hormone (GnRH) agonists and ulipristal acetate, could be implemented as an alternative to surgery. GnRH agonists effectively reduce fibroid and uterine volume, fibroid-related bleeding, and abdominal symptoms. However, their use is limited due to side effects such as hot flashes and increased risk of osteoporosis [3]. Therefore, the only long-term pharmacological treatment of uterine fibroids currently approved is ulipristal acetate, which was first authorized in the European Union in 2012 [4,5,6]. 

Leiomyomas sometimes spread to distant sites, resulting in pulmonary, cardiac, nervous, skeletal, or soft tissue involvement, with the lungs being the most common extrauterine location (80% of cases) [7,8,9,10]. Even though the ability to metastasize is inadvertently a feature of malignancy this uncommon phenomenon is, somewhat paradoxically, described as benign metastasizing leiomyoma (BML). Since its first description by Steiner in 1939, multiple cases have been reported [1,8]. It mainly affects late reproductive-age women [9,11,12]. The mechanism of leiomyoma dissemination remains unclear. The most likely hypothesis postulates hematogenous spread which may occur for example during surgical procedures, but alternatively it may be a result of the coelomic metaplasia similar to some endometriosis cases [8]. Pulmonary benign metastasizing leiomyoma (PBML) is usually an incidental finding on a routine chest X-ray in asymptomatic patients, with multiple nodules in both lungs. However, there are also reported cases of symptomatic patients and solitary masses in just one lung [7,8,9,11]. BML is frequently a diagnostic challenge since other, more common pulmonary lesions, especially cancer metastases have to be excluded in the first place [11,12]. Thus, a vast spectrum of diagnostic tests is frequently applied before fine-needle CT (computed tomography)-guided biopsy or operation, and histopathological examinations are used [7,12,13]. 

We present three cases of BML demonstrating the diagnostic dilemmas, differentials and therapeutic approaches.

## 2. Case 1

**Figure 1:**
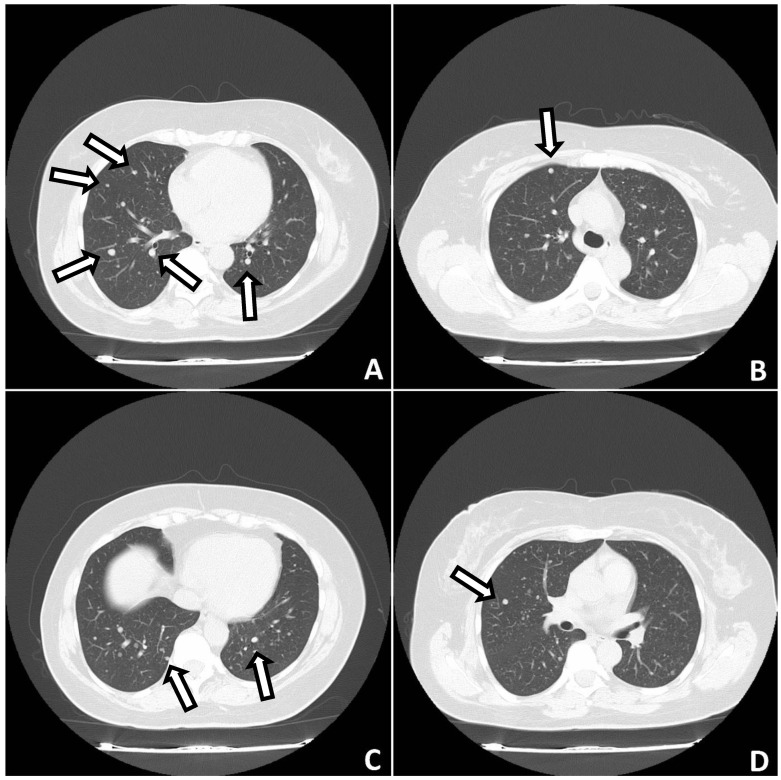
CT of the lungs. Lung nodules are visible in both lungs (arrows) (**A**–**D**).

**Figure 2:**
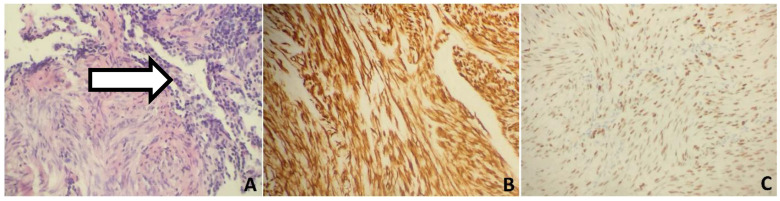
Histology and immunohistochemical analysis of lung nodules found in case 1. (**A**) Histology of one of the lung nodules showed bland spindle cells with no atypical features, no mitotic activity, or necrosis. A bronchioalveolar structure is pointed by an arrow. H&E (Hematoxylin and Eosin), 200×. (**B**) IHC of the nodule with the antibody against desmin, 200×. (**C**) Nuclear staining with ESR1 antibody, 200×.

A 50-year-old female patient with a history of hypertension, thyroid gland tumors, and laparotomy with supracervical hysterectomy due to leiomyoma presented with numerous subcentimeter nodules in both lungs with no pulmonary symptoms. She had never smoked, although radiologic imaging corresponded to advanced neoplastic spread. She had her first period at age 12, and her last at age 38. Her periods had been regular. She was pregnant twice, and she has two children. Her mother suffered from tuberculosis and leukemia. The first discovery of pulmonary changes in this patient was in August 2017 when, during preoperative preparatory examinations before spine surgery, a routine chest X-ray visualized bilateral nodular changes. In October 2017, a chest CT showed subcentimeter nodules in the lungs. Control CT imaging in February 2018 and in June 2018 (Figure 1) confirmed the presence of stable sub-centimeter nodules in both lungs. The results of both examinations were compared: the number and size of the subcentimeter nodules remained the same, no new changes were identified, and the hilar lymph nodes and the mediastinum were not enlarged. Bronchoscopy imaging, the cytology and microbiology of samples taken during bronchial fibroscopy, an ultrasound of the abdomen and supraclavicular lymph nodes, and a lung function tests were all normal. The CEA (carcinoembryonic antigen) serum level result was normal. Therefore, no diagnosis was made. The patient was admitted to the Thoracic Surgery and Transplantation Department of the Pomeranian Medical University in June 2018 and consented to an exploratory anterolateral left minithoracotomy. The pleural cavity was clear, but the lung had numerous hard nodules of various sizes and a larger soft nodule in the lingula of the left lung. The mass in the lingula and a representative nodule from the left lower lobe were extracted and sent for a frozen section and diagnosed as a benign lesion. The postoperative course was uneventful. The final histopathological diagnosis, based on the histological presentation of the obtained material and the fact of hysterectomy due to leiomyoma in the past (12 years prior), was BML of the lung. By immunohistochemistry (IHC), the lesion was negative for CD34, S100, and CD117, and positive for desmin, alpha-smooth muscle actin (ACTA2), estrogen receptor alpha (ESR1), progesterone receptor (PGR). The proliferation index Ki67 was very low (1%) (Figure 2). Two years after the diagnosis, chest CT showed no new changes; the number and size of nodules remained the same. Ultrasound of abdomen and supraclavicular lymph nodes, lung function tests were normal. She had no symptoms. The patient has regular follow-ups without any treatment. 

## 3. Case 2

**Figure 3:**
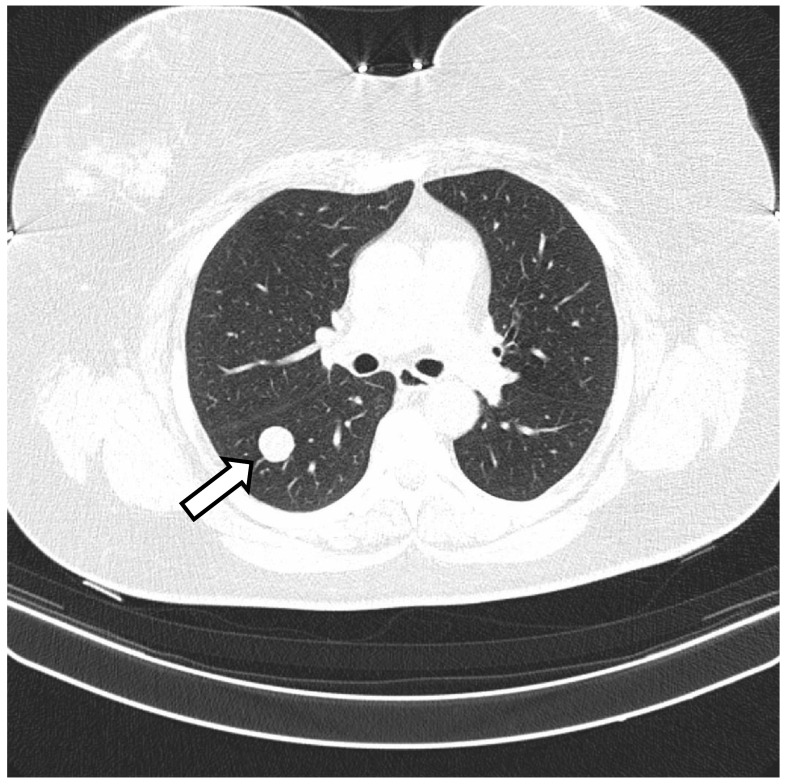
CT of the lungs. A single nodule is observed in the right lung (arrow).

**Figure 4:**
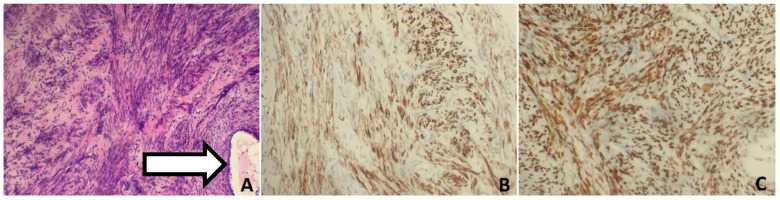
Histology and immunohistochemical analysis of lung nodule found in case 2. (**A**). Histological appearance of the lung nodule with fascicles of uniform spindle cells and no mitotic activity or necrosis. There were entrapped tubules, some dilated, composed of bronchioalveolar epithelial cells (arrow). H&E, 100×. (**B**) IHC staining with desmin antibody, 200×. (**C**) IHC with ESR1 antibody showed nuclear staining, 200×.

A 53-year-old female patient with a history of hypertension and laparotomy with leiomyoma resection presented with a tumor of unclear origin in the right lung. She had never smoked. She had her first period at age 14 and her last period at age 44. Her periods had been regular. She was never pregnant. The mass in her lung was first detected in a routine chest X-ray in June 2018. The presence of a lesion was then confirmed in a chest CT in July 2018 (Figure 3). Liver function test (ALT- alanine aminotransferase, AST- aspartate aminotransferase) results were elevated. CEA serum level was normal. Bronchial fibroscopy revealed copious purulent secretion in the respiratory tract, with the bronchial mucosa swollen and congested. The cytology and microbiology of samples taken during bronchial fibroscopy were normal. Ultrasound revealed an enlarged and fatty liver, thyroid gland tumors, and a 2-cm tumor in the uterus. Lung function testing revealed a disorder of ventilation with a predominance of restriction. The etiology of the mass remained unclear. She was admitted to the Thoracic Surgery and Transplantation Clinic of the Pomeranian Medical University in August 2018 and was qualified for a VATS (Video Assisted Thoracoscopic Surgery) uniportal. Because of one-lung ventilation intolerance, the approach was slightly widened to the standard of the anterolateral mini-thoracotomy. The pleural cavity was clear. The hamartoma-type tumor of segment VI was resected. The intraoperative frozen section confirmed its benign origin. The postoperative course was uneventful. The final histopathological diagnosis, based on the obtained material and the leiomyoma resection in the past (17 years prior), was benign uterine metastasizing leiomyoma of the lung. By IHC the lesion was negative for CD34 and S100, and positive for desmin, ACTA2, ESR1, and Ki67 (2%). Entrapped, cystically dilated bronchioalveolar structures exhibited positive nuclear staining with TTF1 antibody (Figure 4). In September 2018 she had a total abdominal hysterectomy with bilateral salpingo-oophorectomy. The histopathological diagnosis was uterine leiomyomas. At her 2-year follow-up, her condition was stable. Chest CT showed no lesions in the chest, ultrasound revealed enlarged and fatty liver. Lung function testing revealed restriction. The patient’s follow-up is ongoing without any treatment. She was sent to the pulmonology outpatient clinic. 

## 4. Case 3

A 48-year-old patient with a history of an appendectomy at the age of 17, abdominal hysterectomy with bilateral salpingo-oophorectomy due to myoma at the age of 46, right nephrectomy due to oncocytoma at the age of 46, and papillary thyroid cancer necessitating total thyroidectomy and radioactive iodine treatment at the age of 46 presented with numerous nodules in both lungs with no pulmonary symptoms. 

**Figure 5:**
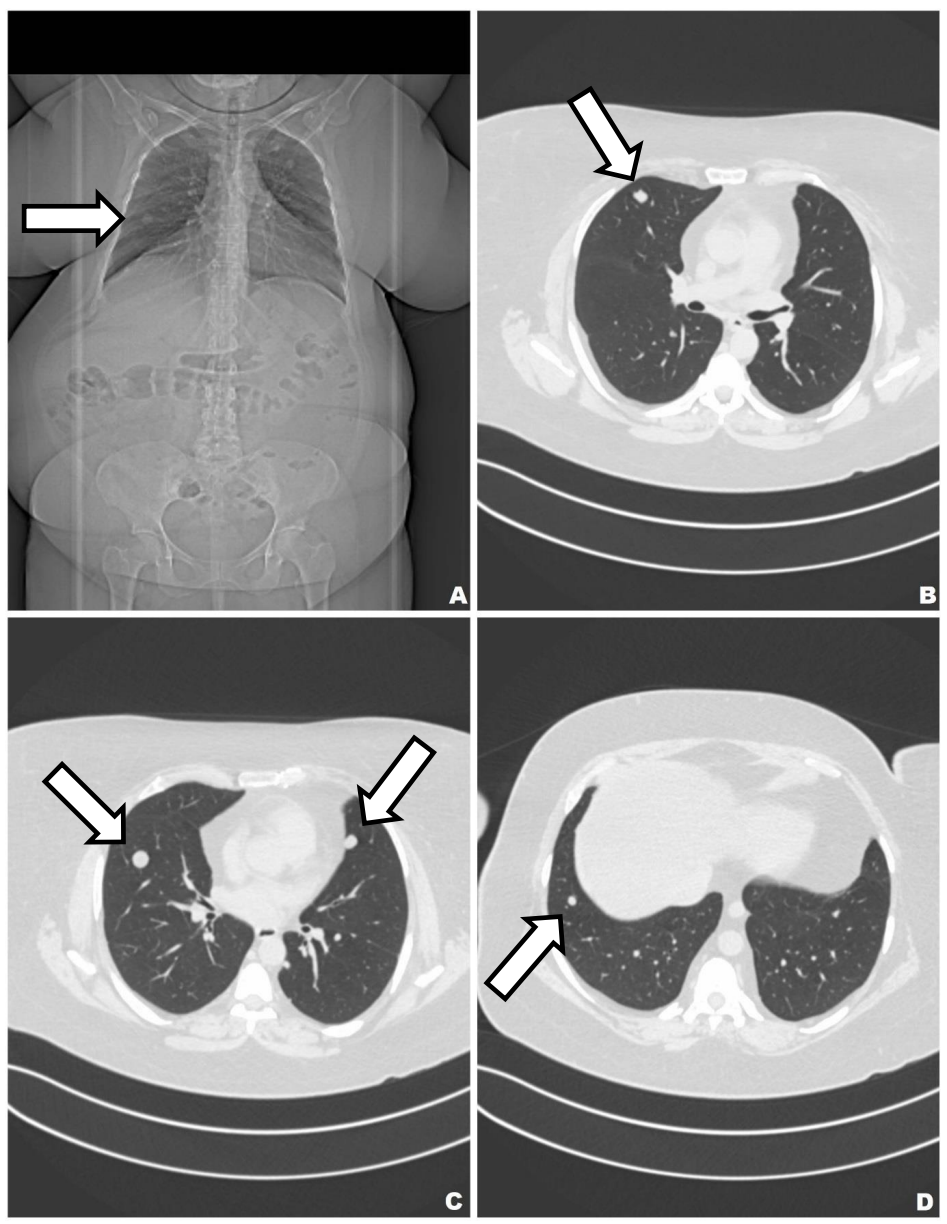
CT of the lung. Multiple cystic nodules are present in both lungs (arrows) (**A**–**D**).

**Figure 6:**
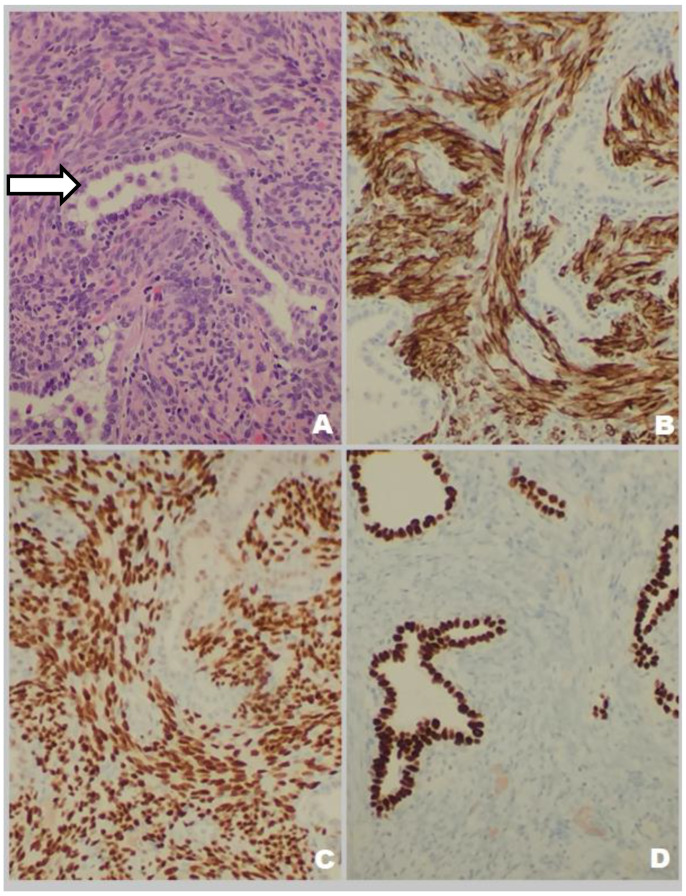
Histology and immunohistochemical analysis of lung nodules found in case 3. (**A**) Histopathology of the lung lesion showing spindle cell fascicles with blunted nuclei and entrapped bronchioalveolar epithelium (arrow) (HE 400×). (**B**) IHC with an antibody against desmin. (**C**) IHC with an antibody against ESR1. (**D**) IHC with an antibody against TTF1 decorating entrapped bronchioalveolar epithelium.

Additionally, she suffered from metabolic syndrome (central obesity, hypertension, dyslipidemia, impaired glucose tolerance, insulin resistance, hyperuricemia), hepatic steatosis, reflux esophagitis, and osteoarthritis of the spine. She has never smoked. Her first period was at 11 years of age and her last was at 46 years. Her periods were regular. She was pregnant twice and she has two children. The first discovery of pulmonary changes in this patient was in January 2018 when, during preoperative preparatory examinations before a hysterectomy with bilateral salpingo-oophorectomy, a chest CT visualized bilateral nodular changes. A suspicious lesion in the liver was discovered on an abdominal ultrasound. Fine-needle aspiration biopsy of the lesion in the liver was conducted. Despite the tests, no diagnosis was made. In April 2019, positron emission tomography with 2-deoxy-2-[fluorine-18] fluoro-D-glucose integrated with computed tomography (18F-FDG PET/CT) showed that the lesions in the lungs were not metabolically active. In addition, there were lesions in the pancreas and one in the left breast with a strong suspicion of neoplastic spread. Chest CT was conducted again in May 2020. The result was compared with the previous chest CT: the size of the biggest nodules remained the same, and the micronodules had become slightly bigger (Figure 5). The result of CEA was normal She was admitted to the Thoracic Surgery and Transplantation Clinic of the Pomeranian Medical University in July 2020, and she was qualified for diagnostic right-sided VATS uniportal type procedure, which was changed to anterolateral mini-thoracotomy. The mass in the middle lobe was extracted and sent for intraoperative histopathological examination. The obtained material was a benign cystic lesion. The postoperative course was uneventful. The final histopathological diagnosis was benign metastasizing leiomyoma (Figure 6). IHC was positive for desmin and ACTA2 as well as ESR1, and negative for podoplanin, CD117, S-100, HMB45 and CD34. The entrapped bronchioalveolar structures were positive for TTF1. At her 6-month follow-up, she has no symptoms. The patient follow-up is ongoing without any treatment. 

## 5. Discussion

Benign metastasizing leiomyomas (BMLs) are relatively uncommon tumors morphologically similar to uterine leiomyomas [14]. At least 161 cases have been described in the literature [8,14]. The condition typically affects women of late reproductive age, particularly during the premenopausal period, with a previous history of surgical management of leiomyomas [15,16]. According to a recent systematic review of the literature, most affected women have a history of myomectomy or hysterectomy at a mean age of 38.5 years and subsequent diagnosis of BML at a mean age of 47.3 years [15]. There have been few studies on the risk factors, related etiology, and clinical behavior of BML [16,17,18,19]. The diagnosis of BML remains challenging, as patients are often asymptomatic, and the mean interval between initial surgery and diagnosis of BML is approximately nine years. However, cases of BML have been identified as early as the time of initial surgery and as late as 31 years postoperatively [20,21,22]. The patients described in this paper had notably different histories. The first patient underwent a supracervical hysterectomy at 38, and BML was diagnosed 12 years later at 50. The second patient had a history of myomectomy at 36, and BML was diagnosed 17 years later at 53. Interestingly, this patient began the menopausal transition at 44, so BML was diagnosed nine years after menopause. BML occurs predominantly within the perimenopausal period, and only a few cases of BML diagnosed after menopause have been described [23]. However, we cannot rule out that the tumor was already present before its incidental finding during a routine chest X-ray. The third patient underwent an abdominal hysterectomy, and lung lesions were identified during preoperative evaluation. In accordance with other authors, in these two patients, the neoplastic changes in these two patients were found years (12 and 17 years, respectively) after the operation for uterine leiomyoma [22]. In the third case, lung lesions were diagnosed before the hysterectomy. In the literature, there are only 10 cases of BML in women with no prior leiomyoma surgery [8]. 

The three cases described in this paper differed in their tumor morphologies; two presented the most common multiple nodule variant, while the other involved a single mass. 

On gross examination, the BML analyzed presented as a well-demarcated solitary lesion or multiple tumors with a whorled cut surface resembling its uterine counterpart. Occasionally a miliary pattern of numerous nodules occupying large areas of the lung parenchyma and leading to respiratory failure was observed [24]. Microscopically, the neoplasm was composed of spindle-shaped cells forming fascicles growing in a whorled pattern. The nuclei were cigar-shaped and uniform. The tumor showed no necrosis or cytological atypia nor a high mitotic index. However, multiple samples should be obtained and carefully evaluated to exclude possible low-grade leiomyosarcoma. Cystic changes and slit- or gland-like spaces lined with bronchial epithelium trapped within the tumor were frequently noted. Previous retrospective analysis of the primary uterine tumor has revealed intravascular growth in some cases, and one study has postulated common pathogenesis of BML and intravenous leiomyomatosis [25]. However, in all three cases, lesions analyzed showed a well-delineated configuration with no intravenous growth or intravenous leiomyomatosis.

IHC markers expressed by BML include ACTA2, ESR1, PGR, desmin, and caldesmon [26,27]. In agreement with these studies, all lung biopsies from the three patients expressed markers of uterine smooth muscle cells, such as ACTA2, ESR1, PGR, and desmin. However, small biopsies may especially hamper the diagnostic process and several spindle cell lesions should be taken into consideration in the differential diagnosis of BML, including Solitary Fibrous Tumor (SFT), gastrointestinal stromal tumor (GIST), inflammatory myofibroblastic tumor (IMT), lymphangioleiomyomatosis, sarcoidosis, leiomyomatous hamartoma, spindle-cell carcinomas, spindle cell melanoma, and metastatic uterine leiomyosarcoma or endometrial stromal sarcoma [28]. Thus, an additional IHC panel was performed including CD10, CD34, CD117, DOG1, ALK, STAT6, cytokeratins, HMB-45, and melanin. CD10 negative reaction excludes SFT or uterine stromal sarcoma metastasis. The absence of CD34 and STAT6 confirms that the lesion is not an SFT. Negative CD117 and DOG1 indicate that this is not a case of no metastatic GIST. Lack of staining for ALK makes a diagnosis of IMT unlikely. Cytokeratin negativity indicates that rule out a spindle cell carcinoma diagnosis can be excluded. Finally, HMB45 and melanin staining excludes melanoma [28]. We retrospectively reviewed the histology of all available postoperative material of the primary lesions in the uteri of all three presented patients. All specimens were classified as typical leiomyoma, the three cases showed the picture of leiomyoma, and no features pointed to the other diagnosis than leiomyoma. Specifically, no features of cellular leiomyoma, atypical leiomyoma, smooth muscle tumor of uncertain malignant potential (STUMP), or intravenous leiomyomatosis were observed. Additionally, there was average cellularity, practically no atypia, no necrosis, and only minimal mitotic activity, with a Ki67 proliferation index lower than 5%, as is frequently observed in leiomyomas. Additional IHC markers were analyzed in case 3, but podoplanin negativity excluded mesothelioma, and bronchioalveolar structures entrapped in the tumor were confirmed with the TTF1 marker. Only in case 3 did one of the diagnosing pathologists decide to perform an extended IHC panel, even though the morphological picture showed no unusual, sinister features. The pattern of staining was like cases 1 and 2. Staining for podoplanin (to exclude mesothelioma), S100, CD117, and HMB45 were negative. TTF1 showed positive staining of the entrapped bronchioalveolar structures.

In all cases, the patients underwent a significant number of tests before a decision to use invasive diagnostics and surgery was made. This is the typical course of events for “yet to be diagnosed” PBML patients: only unspecific clinical features are revealed by the examinations of first resort [22]. 

As for surgical treatment, different approaches were taken. Minimally invasive surgery such as the VATS uniportal approach is currently used worldwide. In the case of the first and third patients, only a few of the multiple subcentimeter nodules were obtained, while in the second patient, the solitary mass of the tumor was resected in toto. The resected material was sent for postoperative histopathological examination, which remains the only method of obtaining the definitive and truly diagnosis [1,7,8,9,11,12,13,14,15,16,17,18,19,20,21,22]. 

The cases described in this paper represent typical patients with BML, given the type of surgery they received in the past. BML has been chiefly described in women after total hysterectomy; only a few case reports describe BML in patients after supracervical hysterectomy. Our first patient had undergone a supracervical hysterectomy, and the second one abdominal myomectomy. In the latter patient, prophylactic total abdominal hysterectomy with bilateral salpingo-oophorectomy was performed two months after the diagnosis of BML and 17 years after the primary surgery. The third patient had an abdominal hysterectomy with bilateral salpingo-oophorectomy after the lung lesions had been found. 

According to the aforementioned literature review, of 161 cases of BML diagnosed worldwide to date, only seven patients had a history of supracervical hysterectomy, and 32 patients underwent a previous myomectomy. Most cases (122) occurred after a total hysterectomy [8]. Nevertheless, the occurrence of metastatic leiomyomas in women after all types of surgeries suggests that any type of uterine surgery predisposes women to their occurrence. One hypothesis that considers surgery as a causal agent for PBML is hematogenous spread occurring during hysterectomy, which is supported by molecular studies demonstrating monoclonality of paired uterine leiomyomas and metastatic lesions [29]. In this sense, many authors believe that an injury during the surgical procedure or necrosis, as well as rupture of the primary lesion in the uterus, promotes the hematogenous spread of the tumor. On the contrary, the peritoneal seeding hypothesis stands that fragments of uterine leiomyoma may implant and proliferate when accidentally left inside the peritoneum after laparotomy or after laparoscopic morcellation. In either case, surgery triggers leiomyoma cell spreading, and my account for cases 1 and 2 from this study. However, the long time between leiomyoma surgery and BML diagnosis argues against these two hypotheses. Alternatively, the metaplasia hypothesis suggests that metaplastic transformation of the coelomic epithelium may occur in almost any place where mesothelial mesenchyme exists. Tumors probably originate from subcoelomic mesenchymal cells, which differentiate into myofibroblasts under the influence of hormonal factors. Therefore, BML is a form of generalized leiomyomatosis with several primary nodules, and estrogen replacement therapy may contribute to such metaplasia [23,29]. This hypothesis may explain the few cases of BML that occurred in women who have never undergone a previous uterine myoma surgery, as the case 3 in our study. Nevertheless, the overall incidence of BML after leiomyoma is unknown, as is the incidence of BML after various types of surgery. Therefore, the specific risks associated with the types of operation cannot be determined. The disease is so rare that it does not seem reasonable to perform a screening test on all women who have undergone uterine surgery for leiomyoma.

No standard management guidelines have been formulated regarding the treatment of BML. Management of BML varies with the presentation pattern and the extent of symptoms. Surgical resection may be the treatment of choice for isolated lesions, and cases with the diffuse disease are more likely to benefit from a systemic approach. Observation, surgical resection, hysterectomy, bilateral oophorectomy, administration of progestins and aromatase inhibitors, and luteinizing hormone-releasing hormone analogs have all been reported as potential treatment options [24,30]. 

In the majority of published cases, benign metastatic leiomyomas express estrogen and progesterone receptors. This is associated with their hormonally dependent growth and spontaneous regression during pregnancy or menopause [9,31]. Evidence for a hormonal influence includes the fact that the pulmonary nodules shrink after menopause, during pregnancy, and after the withdrawal of hormonal contraception, and by the beneficial effects of bilateral oophorectomy [32]. 

Although ulipristal acetate (a selective PGR modulator) is frequently used as an effective treatment for uterine fibroids, its effect on BML is uncertain. Ulipristal acetate reduces the proliferation of leiomyoma cells, remodels the extracellular matrix, and induces apoptosis. It also inhibits gonadotropin secretion and suppresses ovarian function, thus contributing to a hypoestrogenic environment. This induces endometrial hypotrophy and reduces the size of the fibroids. We are aware of three case reports confirming ulipristal acetate as a possible novel non-surgical treatment of BML. All concluded that it might reduce the size of BML nodules. The hypothesis is that ulipristal acetate blocks the progesterone receptors of the lesions, thereby restraining lesion growth [33,34]. In one of the studies, a subsequent CT scan showed an impressive reduction of the lesions [35]. Considering the various characteristics of women diagnosed with BML, treatment should be individualized to each patient. It should depend on the location, the number of metastases, and the hormone receptor status [36,37]. In case of qualification for lung metastasectomy, VATS uniportal approach is recommended if possible. Similarly, as with uterine leiomyoma, surgical treatment and conservative hormonal treatment could be an option for some cases of BML [38,39]. 

## 6. Conclusions

In this paper, we have described three interesting new cases of BPML: two presented with the multiple-nodule variant and one with a single mass. Follow-ups occurred for the three patients after surgery. BPML is rare but should be considered in a differential diagnosis of single or multiple lung nodules, especially among late reproductive-age women with a history of leiomyoma. Histopathological examination is required for final diagnosis and to rule out primary or metastatic malignancy. No standard treatment for BML has been formulated, and treatment should be individualized for each patient. 

## Data Availability

Not applicable.
